# Supplementary material for: From Childhood Residential Green space to Adult Mental Wellbeing: A Pathway Analysis among Chinese Adults
Source: Behav Sci (Basel). 2022 Mar 17;12(3):84. doi: 10.3390/bs12030084 (PMC8945553; doi:10.3390/bs12030084)
Supplement: Supplementary file 1 [file behavsci-12-00084-s001.zip › Supplementary 2.pdf]

Supplemental materials

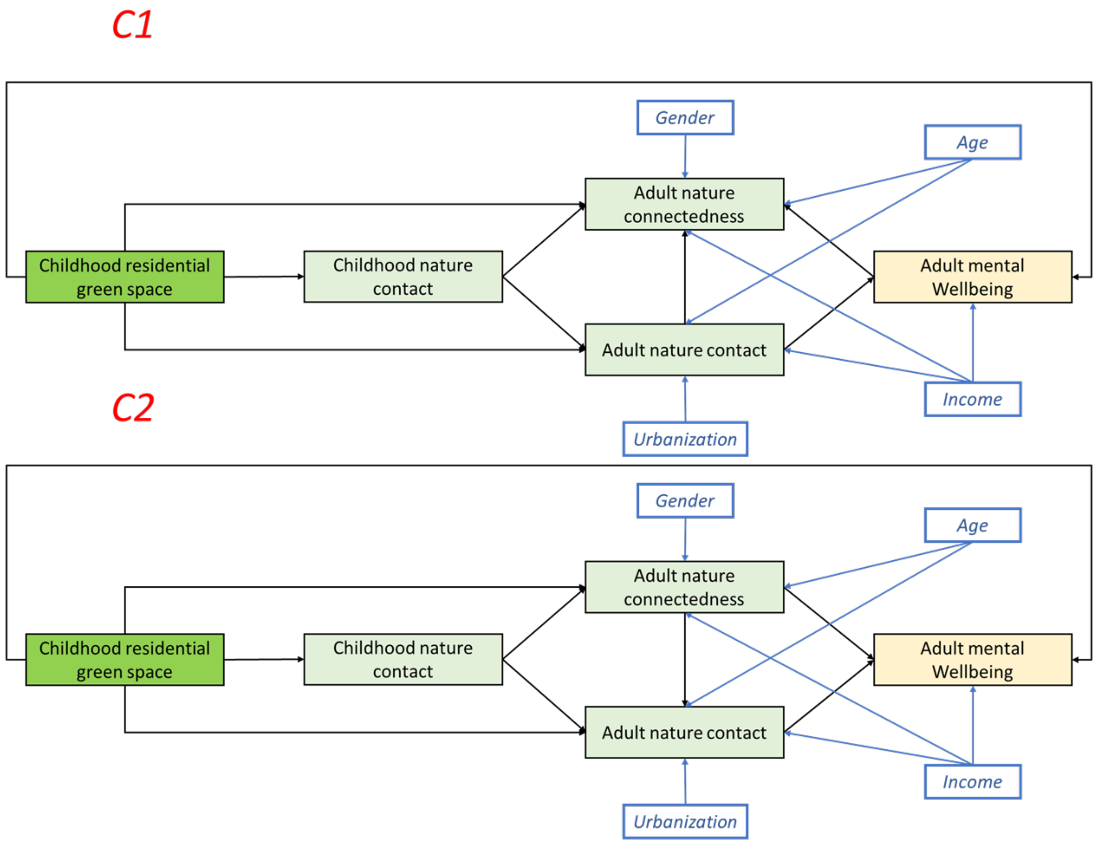

Figure S1. Competing models

Table S1. Model fits of competing models

Model	$\chi 2$/df	GFI	AGFI	CFI	RMSEA	AIC	BIC
Final model	2.97	0.99	0.96	0.98	0.05	99.67	262.30
Model C1	3.17	0.99	0.96	0.98	0.05	101.69	264.31
Model C2	3.40	0.99	0.96	0.98	0.06	104.03	266.65
